# Development of a discrete choice experiment design to explore how patient characteristics influence preferred fixation method in total hip replacement in the UK

**DOI:** 10.1186/s12891-026-10159-7

**Published:** 2026-07-15

**Authors:** Petra Baji, Cecily Palmer, Andrew Moore, Michael R Whitehouse, Jonathan T Evans, Ashley W Blom, Mike Reed, David Sochart, Elsa M R Marques

**Affiliations:** 1 Musculoskeletal Research Unit, Translational Health Sciences, Bristol Medical School, University of Bristol, Bristol, UK; 2 Exeter Hip Unit, Princess Elizabeth Orthopaedic Centre Royal Devon University Healthcare NHS Foundation Trust, University of Exeter, Exeter, UK; 3 Population Health, School of Medicine and Population Health, The University of Sheffield, Sheffield, UK; 4 Trauma & Orthopaedics, Northumbria Healthcare NHS Foundation Trust, University of York, York, UK; 5 Epsom and St Helier University Hospitals NHS Trust, Academic Surgical Unit (ASU), South West London Elective Orthopaedic Centre (SWLEOC), Epsom, UK; 6 Health and Community Sciences, Exeter Medical School, University of Exeter, Exeter, UK

**Keywords:** Implant fixation, Total hip replacement, Discrete choice experiment, Orthopaedic surgeon, Preference

## Abstract

**Background:**

Currently it is unclear how patient characteristics influence orthopaedic surgeons’ decision-making in choice of implant and fixation. The aim of this study was to develop a discrete choice experiment (DCE) design to explore how patient characteristics influence surgeons’ choices of implant fixation in total hip replacement (THR) in a national survey in the UK.

**Methods:**

We followed a three-stage process to develop the DCE design. In stage 1, we used evidence from a literature review, expert panel of five surgeons, and Think-Aloud interviews with two surgeons to develop attributes and levels to describe patient profiles. In the stage 2, we developed a D-efficient experimental design for the survey. In stage 3, we pre-tested the survey using cognitive de-briefing with three surgeons and piloted the survey with ten surgeons.

**Results:**

Final attributes and levels were: age (55,65,75,85 years), sex (female, male), medical condition measured by ASA grade and BMI (3 levels: ASA III and BMI is ≥ 40; ASA III and BMI <40; ASA I or II and BMI < 40), patients’ expected physical activity level after the surgery including sports, everyday activities and employment (mostly inactive, moderately active, very active), and surgeons’ perception of the risk of periprosthetic fracture (high risk: yes, no). We generated an efficient experimental design with 40 patient profiles divided into 4 blocks of 10 profiles to be randomly distributed across respondents. Pre-test of the survey with cognitive debriefing confirmed the feasibility of the DCE task.

**Conclusion:**

We co-developed a DCE design with orthopaedic surgeons to explore how patient characteristics influence THR fixation methods in a survey among UK orthopaedic surgeons. Results of the DCE will inform 1) the design of a large randomised controlled trial (HIPPY) that aims to find out which hip implant fixation (cemented, uncemented, or hybrid) is the most effective and cost-effective for patients aged under 70 years, undergoing primary elective THR and 2) the implementation strategy of trial findings into clinical practice.

**Supplementary Information:**

The online version contains supplementary material available at https://doi.org/10.1186/s12891-026-10159-7.

## Background

Total hip replacement (THR) is a clinically and cost-effective surgical treatment for end-stage osteoarthritis [1]. More than 100,000 primary THRs were performed in the UK in 2019 (National Joint Registry 2020; Scottish Arthroplasty Project 2020) [2, 3]. Approximately half of these were performed in patients under 70 years.

Total hip replacement is made up of prosthetic components that mimic the natural hip joint which is a ‘ball (femoral head) and socket (acetabulum)’ joint. When fitted together the components form an ‘implant’. These components consist of an artificial stem with a ball (femoral head) that is fixed into the patient’s femur, and a cup (acetabular component) that is fixed to the pelvis, into which the ball fits (See Additional File 1 Figure S1). During THR surgery, different ways of fixing the prosthetic stem and cup to the bone can be used. Main types are cemented (the cup and stem components are fixed to the bone with polymethylmethacrylate bone cement), uncemented (the stem and cup are press-fit to the bone and hybrid implants (cemented stem and uncemented cup). These fixation types are associated with different outcomes for the patients (such as risk of revision surgery) and costs for the NHS. Previous research [4] has shown that cemented hip implants are safe, have a long track-record of good revision outcomes, and offer the best value-for-money for men aged over 75 and women aged over 65 years. Getting It Right First Time, an NHS improvement programme designed to improve the quality of care within the NHS by reducing unwarranted variations in practice, recommends that 80% of primary hip replacements in patients aged 70 and over should receive cemented or hybrid prostheses [5]. Evidence for younger patients (under 70 years) is uncertain [6], yet about 75% receive more expensive uncemented or hybrid implants.

Surgeons may have preferences for implants they use in primary elective THR, and it is unclear which factors more strongly influence their decision-making in choice of implant and fixation. It is also unclear how surgeons may change their preferences in relation to patients’ age and sex or other surgical and patient characteristics. Although there are a small number of studies that have identified factors that influence surgeons’ implant choices [7–11], these used structured questionnaire designs in which potential factors were predefined for respondents. Most of these focused on either young patients or those involved in sports with no overarching consensus on implant fixation method [7, 9–11]. Others were conducted in a US or Australian context which may limit their relevance to the UK National Health Service (NHS) system [9, 10].

We are conducting a large programme of research – the Hip Implant Prosthesis Programme for Younger Total Hip Replacement patients (the HIPPY Programme) to find out which hip implants are best for patients with osteoarthritis aged under 70 years. HIPPY comprises a randomised controlled trial (RCT) of cemented, uncemented, and hybrid implants, powered on 10-year revision risks, with a series of complementary studies designed to inform the trial, understand clinical practice, and guide the implementation of evidence. In the first phase, we conduct a national survey of hip surgeons to explore UK practices and factors that determine a surgeon’s preference for implants in patients requiring THR, and to identify surgeons who would be willing to participate in the HIPPY RCT.

This study reports on the development of a discrete choice experiment (DCE) embedded in the national survey that is used to examine how patient characteristics affect surgeons’ choice of fixation technique. We aimed to develop a DCE design that can help to (1) identify main patient characteristics that influence surgeon choice of hip implant fixation method (cemented/uncemented/hybrid); (2) elicit the relative importance of patient characteristics on the choice of fixation method, (3) predict the choice of fixation method for selected patient profiles; (4) calculate which changes in patient characteristics influence choice probabilities, and how; (5) explore observable preference heterogeneity in choices by surgeons’ circumstances such as years of experience, number of operations performed, geographical region); (6) identify the characteristics of surgeons who are non-traders (i.e., have a preference for using a single fixation technic regardless of patient characteristics). We are using the results from the national surgeon survey to directly inform the design of the HIPPY randomised controlled trial and the implementation strategy of trial findings into clinical practice.

## Methods

DCE is a stated preference method, frequently used in health economics to explore patients’ and providers’ preferences for different treatment options, health states or interventions [12–14]. In a DCE, respondents are faced with different hypothetical scenarios of certain goods, services, or health states, from which, they are asked to choose the one they prefer the most. Each different scenario contains different levels of attributes. Responses are then used to elicit the relative importance of these attributes, and to quantify the weights that respondents assign to each level, which can later be used to predict choices, and to understand how priorities are set [14, 15]. See further details of the method described in [16–18].

In this study, we followed a multi-stage approach to develop the DCE design following best practices [19–22]. In stage 1, we developed the attributes and attribute levels based on literature review, expert panel and Think-Aloud interviews with surgeons. In stage 2, we developed the experimental design and the survey. In Stage 3, we used cognitive debriefing to pre-test the survey and piloted the final version with 10 surgeons.

### Stage 1: Development of attributes and levels for the DCE

In the DCE, we use different attributes with varying levels to describe patient profiles. We applied a simplified version of the three-step attribute development approach suggested by Johansson et al. [19]. In the first step, we used the 20th Annual Report 2023 of the National Joint Registry combined with a systematic literature review [23] to identify an initial list of patient characteristics and levels that potentially influence surgeon choice of implant fixation (See in Table S1 in Additional file 1).

In the next step, we invited an expert panel of five orthopaedic surgeons (MRW, AWB, JTE, MR, DS) to give their opinion the initial list of attributes. They were also asked to be involved in the development of a meaningful description of the attributes and levels. Discussion was carried out via several rounds of group emails in June 2023 moderated by PB and EMRM, where surgeons got the initial list of attributes and levels with a set of questions to reflect on (See questions in Supplementary material 1 in Additional file 1). Surgeons were able to see and reacted to each other’s replies and moderators could ask clarifying questions if it was necessary. After saturation point was reached (when no new views were added) we made the necessary adjustments in the attributes and levels.

In the third step, using these modified attributes and levels, we developed an example choice set with 10 patient profiles using the “dcreate” command of Stata 17.0 (StataCorp LLC) and conducted Think-Aloud interviews with two surgeons (see an example for a choice set in Supplementary material 2 in Additional file 1 ). The interviews were conducted by CP in September 2023 using Microsoft Teams during which surgeons were asked to complete the DCE whilst under observation, and to share with the interviewer comments and questions that arose relating to DCE wording and experience of completion. The interviewer also prompted for further information and clarification where required. After the interviews, we made the necessary adjustments in the list and wording of attributes. The final version was approved by the members of the expert panel.

All experts and interview participants consented to be involved in the study.

### Stage 2: Development of the experimental design and the survey

Our DCE model builds on a model developed by de Bekker-Grob et al. [24]. The DCE contains three choice alternatives in each task (i.e., cemented, uncemented or hybrid), and all attribute levels are treated as scenario variables.

The model builds on the random utility maximisation framework [25]. We assume that respondents derive utility from each alternative in the choice set and choose the alternative with the highest utility. Utility comprises a deterministic component (observable to the researcher) and a random component (error term) [26]. In the multinomial logit model, these error terms are assumed to be independently and identically distributed with a Gumbel extreme value distribution [25]. Deterministic parts of the utility function attached to each choice option can be written as the linear combination of respondent’s alternative specific preferences ($\:{\beta\:}_{k}$) and attributes’ levels used to describe patient profiles ($\:{X}_{k}$) [27]:

$$\:{V}_{cemented}=0$$

$$\begin{aligned}\:{V}_{uncemented}&={\beta\:}_{1}+{\beta\:}_{2}Age+{\beta\:}_{3}Male\:\\&+{\beta\:}_{4}MedicalCond1+{\beta\:}_{5}MedicalCond2\\&+{\beta\:}_{6}ActivityModerate+{\beta\:}_{7}ActivityVActive\\&+{\beta\:}_{8}Fracturerisk\:\end{aligned}$$

$$\begin{aligned}\:{V}_{hybrid}&={\beta\:}_{9}+{\beta\:}_{10}Age+\:{\beta\:}_{11}Male\\&+{\beta\:}_{12}MedicalCond1+{\beta\:}_{13}MedicalCond2\\&+{\beta\:}_{14}ActivityModerate+{\beta\:}_{15}ActivityVActive\\&+{\beta\:}_{16}Fracturerisk\end{aligned}$$

$\:V\:$represent the systematic utility stated preference that a respondent has for a fixation method with the cemented alternative is set to 0.

$\:{\beta\:}_{1}and\:{\beta\:}_{9}\:$represent alternative specific constants for a fixation method, option cemented acts as the reference level.

$\:{\beta\:}_{2\:}-\:{\beta\:}_{8\:}and\:{\beta\:}_{10\:}-\:{\beta\:}_{16\:}$represent alternative specific parameter or coefficients for attribute(level)s.

The value of each coefficient represents the importance that surgeons assign to a certain patient characteristic, and (one or two sided) t-tests of the parameters can tell if these are significantly different from 0, i.e., if these influence the choice of fixation method. The sign of the coefficient reflects whether the attribute (level) has a positive or negative effect on the choice of a specific implant type [24].

Using this model, we generated a D-efficient experimental design with 40 patient profiles with a multinomial logit model using Ngene software (Version 1.3.0, ChoiceMetrics, available at: https://choice-metrics.com/index.html). The design was optimised for D-efficiency by minimising the D-error of the design. This method is used to select combinations of attribute levels from the full fractional design (all combinations of attribute levels), to achieve a reduced number of choice-sets manageable for respondents, but still be able to efficiently obtain the most information from the responses to ensure more precise parameter estimates [28]. Only main effects were used and the following constraints were applied: ‘very active’ activity level could not appear together with 85 years of age or with medical condition ‘ASA III & BMI > 40’. We used non-informative priors to generate the profiles (with only the expected sign of certain attributes were taken into account). To reduce the burden on respondents, the 40 profiles were grouped into 4 blocks, with each block containing 10 patient profiles.

Given that we do not have informative priors on the parameter values in our utility functions, the required sample size for a given power and significance level cannot yet be determined. Nevertheless, the rule of thumb as proposed by Johnson and Orme [29] suggest that with our design, and with 10 choice sets per respondents, we can get reliable estimates of the parameters with about a hundred surgeons completing the DCE task.

The final DCE design was then embedded in the national survey. The survey started with structured questions on surgeons’ practices such as years in practice; volume of primary elective hip replacements performed over the previous year; characteristics of their patients and hospitals in which THRs were performed; preferred choice of hip implant fixation and type. The second part of the survey comprised the DCE task. In the third part, we added further questions on the factors that are influencing implant choices. Specifically, we asked surgeons to score 17 different factors that potentially influence their choice of fixation method for their patients using a 4-point scale Likert-scale (never an influence, rarely an influence, sometimes an influence, always an influence) and to add any factors that are missing from the list. The final survey tool was developed on Qualtrics platform that enables randomisation of the four blocks of choice sets between respondents.

Stage 3: Cognitive de-briefing of the survey and pilot.

Cognitive debriefing was done via e-mail. We contacted three surgeons from the network of the expert panel and asked them to fill in the survey and provide their feedback. After the survey was finalised, we piloted it with 10 orthopaedic surgeons for a final check for feasibility and validity of the questionnaire and DCE data before launching it.

## Results

Development of attributes and attribute levels.

### Initial list of attributes and the expert panel

The following patient characteristics were identified from the NJR report and the literature as potential factors that can influence choice of fixation technique: sex, age, BMI, expected activity level of the patient after the surgery, medical condition, and osteoporosis. Unsing these domains, an initial list of attributes and levels were developed (See Additional file 1 Table S1) for the expert panel.

The expert panel agreed that all six patient characteristics were relevant and could potentially influence implant choice. Discussion of each attribute is summarised as follows:

#### Sex

It was agreed that men are more likely to receive an uncemented implant than cemented or hybrid [2], although the reason for this could not be clearly explained. The expert panel discussed whether the study should describe sex or gender of the patient. It was agreed to use sex as an attribute rather than gender as it aligns with the most recent NJR data collection processes, and recommendations from the Office of National Statistics [20].

#### Age (patient ≤ 70 or > 70 years old)

Evidence from a systematic review of RCTs showed that cemented implants have improved Harris hip scores at 0–2 and 2–10 years from surgery [6], and may be cost-effective for men over 75 years and women over 65 years [4]. Evidence is unclear for younger patients, and these are more likely to receive hybrid or uncemented implants [2]. An objective of our DCE study is to determine how age influences surgeons’ choice of implant fixation method and at which age cut-off surgeons are more likely to change their preferences. The expert panel clarified that this was the reason for including patients over 70.

#### BMI

It was also agreed that BMI of the patient is likely to be considered when choosing between implant types as it can affect surgical exposure, operative complexity, and postoperative risk. Higher BMI levels, particularly severe obesity (BMI ≥ 35–40), can further constrain implant choice: surgeons suggested that cemented fixation might be less commonly selected in this group because the cementing process is technically more challenging (e.g., maintaining limb position during cement curing), and in some cases robotic surgery (where available) might be preferred in these patients, which is typically limited to uncemented or hybrid implants.

#### Medical condition

Surgeons in the expert panel agreed that the overall medical status and preoperative risk of patients influence their decision about implant fixation types, particularly when balancing operative complexity with the likelihood of complications. There was consensus that the American Society of Anaesthesiologists physical status classification system (ASA grade) [30] - in line with the NJR terminology - is suitable to describe risk of medical problems.

#### Expected activity level of the patient after the surgery

We define activity level as the patients’ expected physical activity level after the surgery. The expert panel suggested that physical activity should include sports, everyday activities and employment role. Surgeons in the expert panel agreed that there may be a preference for uncemented or hybrid implants for patients who are more active. To define attribute levels, we decided to use The University of California, Los Angeles (UCLA) activity score system [31] – this system is widely used for assessing activity levels in patients having joint replacement [32]. The expert panel suggested that distinguishing between three activity levels would better capture real life decision-making situations than simply distinguishing between more active and less active patients. The final three attribute levels therefore include: 1) mostly inactive - covering UCLA levels 2–3, 2; moderately active - covering UCLA levels 4–6, and 3; very active - covering UCLA levels 7–10.

#### Osteoporosis/bone quality

Surgeons in the expert panel agreed that bone quality influences decisions on implant fixation methods. Surgeons agreed that it is the perceived risk of intra- or post-operative periprosthetic fracture that matters the most, which often goes beyond the formal diagnosis of osteoporosis, and it is a subjective consideration of age, sex, and any previous fracture history (particularly hip, lumbar or wrist). As such, the panel preferred to use the attribute of risk of periprosthetic fracture rather than diagnosis of osteoporosis. They also formulated the definition for this attribute.

Employment and bone quality were discussed as potential additional attributes that can influence fixation choice. It was debated whether employment should be included as a separate attribute, with mostly physical labour that is affecting implant choices. Experts agreed that it is sufficient to incorporate employment in the physical activity attribute with specifying this in the definitions of attribute levels. Surgeons considered that decision making is often informed by radiographic imaging of their patients, but the panel also agreed that asking surgeons to interpret X-ray images (radiographs) would create an undesirable complexity to the DCE task. It was also discussed that patient anatomy (such as acetabular anatomy or bone loss and on the femoral side by the shape of the intramedullary canal) would be difficult to translate into an attribute. The possible application of the Dorr classification system [33] was also debated (as patients with Dorr A femurs are often not good candidates for uncemented prostheses), but its familiarity and use in clinical practice was questioned and therefore not included in the final set of attributes.

We incorporated the proposed changes in the list of attributes, their levels and definitions, and these modifications were used to create an example choice set for the Think-Aloud interviews (see an example for a choice set in Supplementary Material 2 in Additional file 1).

### Think-aloud interviews

The two Think-Aloud interviews were conducted in September 2023. Feedback from these interviews showed that medical status and BMI are highly correlated (for example with cases BMI ≥ 40 being considered ASA III classification or higher). We therefore combined these domains in the final version of the designinto a single attribute: Risk of medical problems before or after surgery & BMI, and used the following three attribute levels: (1) at higher risk of medical problems before or after Surgery (ASA III) and BMI is 40 or greater, (2) at higher risk of medical problems before or after Surgery (ASA III) and BMI less than 40, (3) not at higher risk of medical problems before or after Surgery (ASA I or II) and BMI less than 40. To be able to create meaningful levels, the study team also decided that we focus on patients with ASA Grades I-III (define here) as patients with ASA Grade IV or V (patients with a severe systemic disease that is a constant threat to life) are less likely to undergo elective THR or to do so for reasons that are not generalisable to the majority of patients in the age group of interest for this programme of work.

Think-Aloud interviews also draw attention to unrealistic combinations of attribute/levels in patients. Thus, we applied the following constraints in the DCE design: ‘Very active’ activity level would not appear together with medical condition ‘ASA Grade III and BMI ≥ 40’ or with age of ‘85 years old’ to ensure that more realistic patient profiles are presented to surgeons.

The study team also decided to include four attribute levels for age instead of two (*≤ 70 or > 70 years old)* in the final design using specified ages: 55 years old, 65 years old, 75 years old and 85 years old, to be able to find a cut-off where surgeons are more likely to change their preferences. The final list of attributes and levels are presented in Table 1.

**Table 1:** Final list of attributes and levels used in the DCE

Attribute	Levels and definitions	
Sex	Female	
	Male	
Age	55 years old	
	65 years old	
	75 years old	
	85 years old	
Medical condition and BMI	At higher risk of medical problems before or after Surgery (ASA III)	BMI 40 or greater
	At higher risk of medical problems before or after Surgery (ASA III)	BMI less than 40
	Not at higher risk of medical problems before or after Surgery (ASA I or II)	BMI less than 40
Expected physical activity level of the patient after the surgery (including sports, everyday activities and employment)	Mostly inactive: Wholly or mostly inactive, or only participates in mild activities such as walking, limited housework, and limited shopping	
	Mostly active: Participates in mild to moderate activities such as walking, swimming, can do unlimited housework or shopping, and not doing heavy physical labour	
	Very active: Participates in active events such as bicycling, bowling and golf or impact sport such as jogging, tennis, skiing, and/or doing heavy physical labour	
Perceived high risk of periprosthetic fracture	Yes: Either suggestion of osteoporosis, significant bone loss, or prior history of any bone fracture	
	No: No suggestion of osteoporosis, no or mild bone loss, no prior history of any bone fracture	

### Experimental design and construction of choice sets

We generated a D-efficient experimental design with 40 patient profiles from all possible combination of attribute levels and grouped them into four of 10 patient profiles to be randomly presented to respondents in the survey. The experimental design is presented in Supplementary materials Table S2.

An example of a choice set is presented in Table 2. The DCE task started with a description of a hypothetical scenario as follows: “Please consider the following patient. Please assume that this patient has osteoarthritis and that osteoarthritis is the indication for surgery.” After presenting the patient profile, surgeons are asked which fixation method would be their most preferred option to use for this patient, with three choice alternatives: (1) a cemented construct, (2) an uncemented construct and (3) a hybrid construct. The surgeon is then asked, based on these attributes alone, which fixation types would they be willing to randomise the patient to, if they were part of a trial in which this patient could receive a construct with a randomly assigned fixation, where bearing surface and other surgical characteristics would be left to their choice.

**Table 2:** Example for a choice set

**Patient profile 1/10:** Please consider the patient: Please assume that this patient has osteoarthritis and that osteoarthritis is the indication for surgery.	
**Patient 1**	
Sex	Female
Age	75 years old
Risk of medical problems before or after surgery & BMI	At higher risk (ASA III) & BMI < 40
Expected physical activity level of the patient after the surgery (including sports, everyday activities and employment)	Moderately active*
Perceived high risk of periprosthetic fracture (suggestion of osteoporosis, significant bone loss, or prior history of any bone fracture)	Yes
*Participates in mild to moderate activities such as walking, swimming, can do unlimited housework or shopping, and not doing heavy physical labour	
Which fixation method would be your most preferred option to use for this patient? ○ A cemented construct ○ An uncemented construct ○ A hybrid construct	

### Cognitive de-briefing and pilot

Cognitive debriefing with 3 surgeons confirmed that the DCE task was understandable, with no difficulties reported. Thus, it was not necessary to make any changes in the design or wording of the DCE task.

The final pilot of the questionnaire with 10 orthopaedic surgeons confirmed that the length of the questionnaire was manageable (could be completed in approximately 10 min). Final check of the pilot data in December 2023 confirmed the feasibility and validity of the questionnaire and DCE design.

## Discussion

In this study, we have developed a DCE experiment design for a survey among orthopaedic surgeons across the UK to explore how patient characteristics are influencing choice of fixation method when performing THR. We found that the DCE method can be applied for this purpose and is a feasible method to be used in this kind of survey.

We co-developed the designs with orthopaedic surgeons making sure that the attributes cover the most relevant patient characteristics and real-life choices they make on an everyday basis. Think-Aloud interviews, using an example DCE choice set, further refined the attributes and the design by drawing attention to the interrelation between attributes (BMI and medical condition) and implausible combinations of attribute levels which we avoided in the final design. Cognitive debriefing confirmed that the final design was feasible, and the task was manageable and could be completed in a short time (10 min) which was an important expectation to the survey considering the busy schedule of orthopaedic surgeons.

We followed a multi-stage process recommended by best-practices to develop the attributes and the design. Surgeons (the target population) was involved in all stages of the development. Nevertheless, the number of surgeons involved in the development was still low, and we will not expect to receive much more than 100 responses in the national survey. Nevertheless, the DCE of this size will still be useful to better understand the drivers of surgeons’ fixation choices.

### Comparison to previous literature

We are not aware of any previous study using a DCE methodology to understand surgeons’ implant choices and preferences. However, the development process provided reassurance that this technique is fit for the purpose of the study. Previous studies on fixation choices using structured questionnaire designs also identified that age and activity level were important influences of fixation choices. For example, an Australian study found that orthopaedic surgeons preferred uncemented fixation for younger patients [10]. Another study among the members of the British Hip Society (*n* = 109) found that uncemented implants were the most preferred choice for patients engaged in active sports closely followed by hybrid implants [7]. Registry data from the National Joint Registry shows differences in fixation choices in terms of sex of the patient (male patients seem more likely to receive an uncemented implant than cemented or hybrid [34]), however the reason for this is not clear. Evidence for other attributes selected such as health status, BMI and fracture risk are also more anecdotical and less documented in the scientific literature.

### Limitations

We co-developed the survey and DCE design with orthopaedic surgeons to ensure that the choices presented reflected real-world decision making, following a multi-stage process aligned with best-practice recommendations. Nonetheless, the development of a DCE is subject to several limitations.

It is possible that we did not capture certain clinically important attributes—such as patient anatomy, bone quality, or bone morphology and geometry (Dorr femoral classification, femoral canal morphology, acetabular bone stock, deformity, and underlying disease etiology) —that may meaningfully influence surgeons’ decisions. In addition, some attributes (for example, age) may function as proxies for underlying factors like perceived bone quality.

Additionally, fixation methods were broadly categorised as cemented, uncemented, or hybrid constructs. This classification may oversimplify clinical decision-making by failing to capture the diversity of implant designs (e.g. uncemented stems differ in collar presence, coating extent, and mode of biological engagement) and bearing surfaces (e.g. ceramic-on-polyethylene, ceramic-on-ceramic, metal-on-polyethylene, and dual-mobility articulations). These factors may interact with both fixation choice and patient characteristics; thus, our approach may not fully reflect the complexity of real-world practice and may overlook important nuances in surgeon preference.

The importance of factors other than patient characteristics (such as clinical guidelines, personal audit of practice, peers and mentors, published evidence, institutional procurement policies, patient preferences) were not included in the DCE design, but their importance will be explored by a ranking exercise in the same survey.

The absence of priors during design generation also limits the efficiency of the DCE design. Finally, we restricted the design to main effects only, although interactions between attributes (such as age and activity level) may also play a role in shaping preferences.

### Future research and analysis

After data collection is complete, we will estimate a multinomial logit model using choice data from the survey, and we will predict the choice probabilities for the three fixation methods for some selected typical patient profiles. We will then calculate the effects of changing patient characteristics on the choice probabilities. This way we will ensure that DCE results are well understandable for both clinicians, decision makers, and to inform the HIPPY RCT.

We will also explore observed heterogeneity in preferences using surgeon characteristics as interaction terms with the alternative specific constant in the regression model. We expect that the sample size will not be sufficient to reliably estimate more advanced models that account for unobserved heterogeneity such as mixed logit or latent class models.

We will also explore the share and characteristics of non-traders, i.e., those who choose the same fixation method in each choice set regardless of patient characteristics. These results on heterogeneity and non-traders will help the implementation of the HIPPY trial findings into clinical practice.

The analysis will be conducted in Apollo R package (http://apollochoicemodelling.com/index.html). Goodness of fit measures such as log-likelihood, McFadden’s R^2^, the Akaike and Bayesian information criteria [26] will be calculated and presented.

## Conclusion

Understanding why and how surgeons choose implants for their primary elective hip replacement patients will help to understand current practices and will help hospital and clinical decision makers in their procurement decisions. The results from this DCE study will also inform the design of a large RCT, HIPPY, by randomising patients between cemented, uncemented, and hybrid to find out which type is most effective and cost-effective and will aid in the implementation of the trial findings.

## Supplementary Information

Supplementary Material 1.

## Data Availability

Data sharing is not applicable to this article as no datasets were generated or analysed during the current study.

## Abbreviations

ASA grade: American Society of Anaesthesiologists physical status classification system
DCE: Discrete choice experiment
UCLA activity score system: The University of California, Los Angeles (UCLA) activity score system
THR: Total hip replacement
