# Supplementary material for: Development of a discrete choice experiment design to explore how patient characteristics influence preferred fixation method in total hip replacement in the UK
Source: BMC Musculoskelet Disord. 2026 Jul 15;27:857. doi: 10.1186/s12891-026-10159-7 (PMC13656620; doi:10.1186/s12891-026-10159-7)
Supplement: Supplementary file 1 [file 12891_2026_10159_MOESM1_ESM.pdf]

Supplementary materials

Development of a discrete choice experiment design to explore how patient characteristics influence preferred fixation method in total hip replacement in the UK

Petra Baji, Cecily Palmer, Andrew Moore, Michael R Whitehouse, Jonathan T Evans,

Ashley W Blom, Mike Reed, David Sochart Elsa M R Marques

Figure S1 Total Hip Replacement

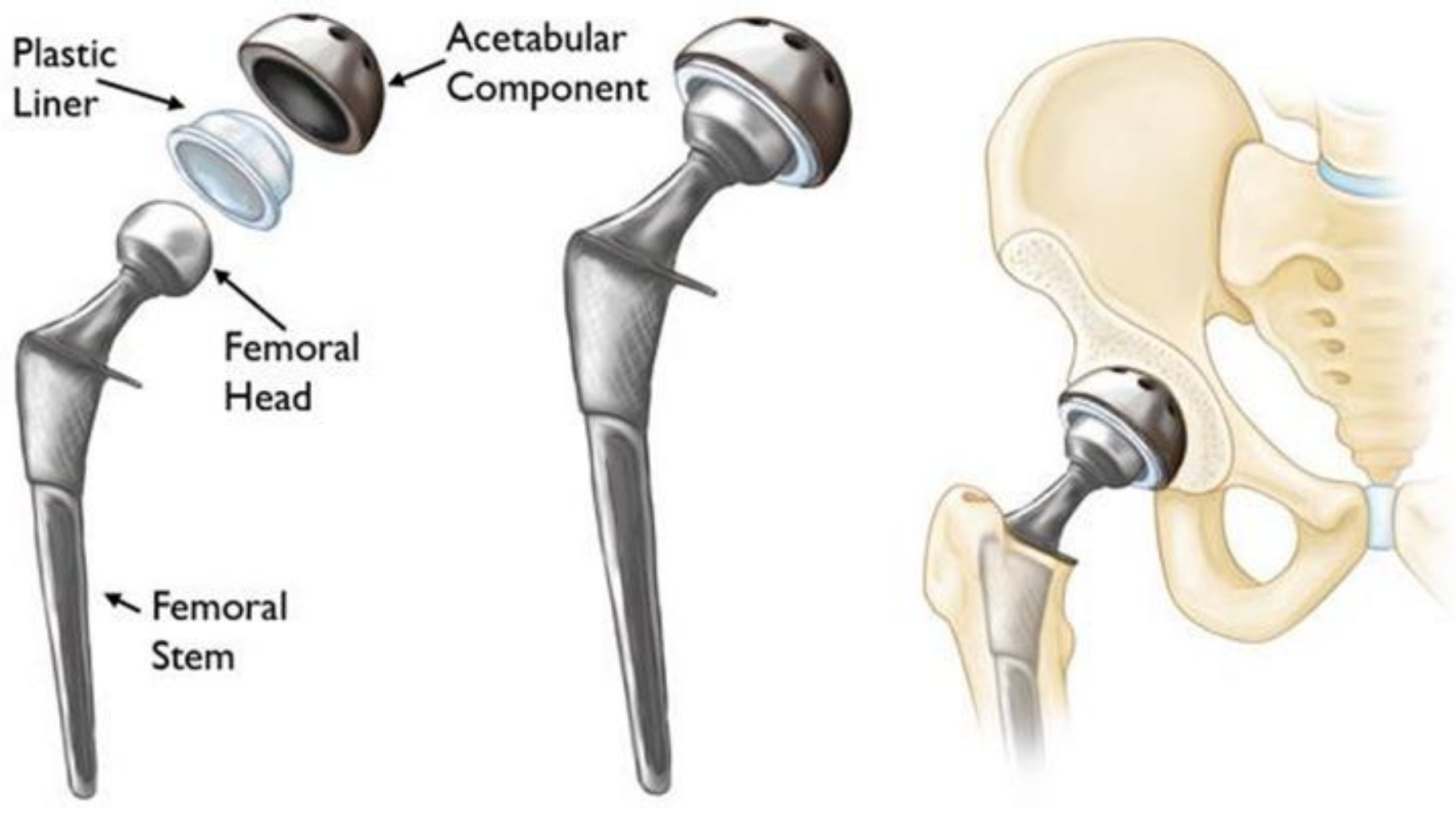

Image: Total Hip Replacement - OrthoInfo – AAOS - https://orthoinfo.aaos.org/en/treatment/total-hip-replacement/

Table S1 Initial list of attributes used in the DCE

Attribute	Level	Definition
Sex	Male	
	Female	
Age	≤70	
	>70	
Severely Obese (BMI of 35 or greater)	Yes	
	No	
Expected activity level of the patient after the surgery	Mostly inactive	Mostly inactive or sometimes participates in mild activities such as walking, limited housework, and limited shopping (correspond to UCLA scores 2-3)
	Active	Capable of participating in mild or moderate activities, can do unlimited housework or shopping (correspond to UCLA scores 4-6)
At higher risk of medical problems before or after Surgery (ASA 3+)	Yes	ASA grade I and II
	No	ASA grade III, IV or V
Osteoporosis	Yes	Diagnosed with osteoporosis
	No	No diagnoses of osteoporosis

Supplementary Material 1: Initial list of questions to the Expert Panel on the Initial list of attributes used in the DCE

We would greatly appreciate if you could briefly answer these 5 questions on the attributes we have selected to describe patient profiles:

(please see the DCE task and attributes below the email)

1. Do you think all 6 attributes (age, sex, BMI, Activity level, ASA grade, OP) are relevant and potentially influence the choice of fixation method? If not, why?
2. Do you think we missed out some important patient characteristic that might influence the choice? If yes, what are those?
3. Do you think sex have an influence on the choice of fixation method on its own, i.e. not thorough osteoporosis? Shall we include sex?
4. Is osteoporosis the best indicator for bone quality? If not, what would you suggest instead?
5. We aim to distinguish between two activity levels “Mostly inactive” (UCLA 2-3) vs “Active” (UCLA 4-6) to capture the intensity of the implant use. We suggest to use the UCLA activity scale (see below) to define the two states. What do you think which levels from the list would best capture the “Active” state (where implant is in intense use), while being still realistic? Are levels 7-10 are out of question for patients with hip replacement?
    1. Wholly inactive: dependent on others; cannot leave residence
    2. Mostly inactive: very restricted to minimum activities of daily living
    3. Sometimes participates in mild activities such as walking, limited housework, and limited shopping
    4. Regularly participates in mild activities
    5. Sometimes participates in moderate activities such as swimming and can do unlimited housework or shopping
    6. Regularly participates in moderate activities
    7. Regularly participates in active events such as bicycling
    8. Regularly participates in very active events such as bowling or golf
    9. Sometimes participates in impact sports such as jogging, tennis, skiing, acrobatics, ballet, heavy labor, or backpacking
    10. Regularly participates in impact sports

Supplementary material 2: The hypothetical scenario and an example for a choice task used in the Think-Aloud interviews

Choice experiment

This section will present ten patient profiles. Please assume that all patients have oesteoarthritis and that oesteoarthritis is the sole indication for surgery.

We would like you to consider the attributes that are used to describe patients. We will then ask you your preferred fixation, and the range of fixation options you would be willing to use for each patient profile.

Attribute	Level	Definition
Sex	Male	
	Female	
Age	≤70	
	>70	
Severely Obese<br>(BMI of 35 or greater)	Yes	BMI of 35 or greater
	No	BMI less than 35
Expected physical activity level of the patient after the surgery (including sports, everyday activities and employment)	Inactive or mostly inactive	Wholly or mostly inactive, or only participates in mild activities such as walking, limited housework, and limited shopping
	Moderately active	Participates in mild to moderate activities such as walking, swimming, can do unlimited housework or shopping, and not doing heavy physical labour
	Active	Participates in active events or impact sports, and/or doing heavy physical labour
At higher risk of medical problems before or after Surgery (ASA 3+)	Yes	ASA grade III, IV or V
	No	ASA grade I and II
Perceived high risk of periprosthetic fracture	Yes	Either suggestion of osteoporosis, significant bone loss, or prior history of any bone fracture
	No	No suggestion of osteoporosis, no or mild bone loss, no prior history of any bone fracture

Patient profile 1:

Please consider the patient:

Please assume that this patient has oesteoarthritis and that oesteoarthritis is the sole indication for surgery.

**Patient 1**	
**Sex**	Male
**Age**	>70
**Severely Obese** (BMI of 35 or greater)	Yes
**Expected physical activity level of the patient after the surgery** (including sports, everyday activities and employment)	Inactive or mostly inactive
**At higher risk of medical problems** before or after Surgery (ASA 3+)	Yes
**Perceived high risk of periprosthetic fracture** (suggestion of osteoporosis, significant bone loss, or prior history of any bone fracture)	Yes

Which fixation method would be your most preferred option to use for this patient?

- A cemented implant
- An uncemented implant
- A hybrid implant

Table S2 Experimental design

Block	Card number	Age<br>0= 55 years old<br>1 = 65 years old<br>2 = 75 years old<br>3 = 85 years old	Male<br>0=Female<br>1=Male	ASA grade & Obesity<br>0 = Not at higher risk (ASA I or II) & BMI < 40<br>1 = At higher risk (ASA III) & BMI < 40<br>2 = At higher risk (ASA III) & BMI ≥ 40	Activity level<br>1 = Mostly inactive<br>2 = Moderately active<br>3 = Very active	Fracture risk<br>0 = No<br>1 = Yes
1	1	2	0	1	1	1
1	2	2	0	0	0	1
1	3	0	0	0	2	0
1	4	2	0	1	2	1
1	5	2	0	0	1	0
1	6	1	1	1	1	0
1	7	2	0	0	2	0
1	8	1	1	2	1	1
1	9	3	1	2	0	0
1	10	0	1	2	0	0
2	1	2	1	1	2	1
2	2	0	1	1	1	1
2	3	0	1	2	1	1
2	4	3	0	2	1	0
2	5	3	1	0	0	1
2	6	2	0	2	0	0
2	7	3	0	0	1	1
2	8	0	1	2	0	1
2	9	0	0	0	1	1
2	10	1	1	0	0	1
3	1	1	1	0	1	0
3	2	3	1	1	0	0
3	3	1	0	1	2	1
3	4	2	1	1	0	0
3	5	2	1	2	1	0
3	6	1	1	1	2	0
3	7	1	0	2	0	1
3	8	3	1	0	1	0
3	9	3	0	1	1	0
3	10	0	0	1	2	0
4	1	2	1	0	2	1
4	2	0	0	1	0	1
4	3	3	0	2	1	1
4	4	0	0	0	0	0
4	5	1	1	0	2	0
4	6	3	0	2	0	1
4	7	1	1	0	2	1
4	8	0	0	2	1	0
4	9	1	0	1	0	0
4	10	3	1	1	1	1

Note: D-error or the final design was 0.497238. The generation of the design was stopped after 101,081 evaluations representing 35,500 iterations of swapping.
